# Gastrointestinal basidiobolomycosis presenting as an inflammatory colonic pathology: management challenges

**DOI:** 10.1093/jscr/rjag329

**Published:** 2026-07-20

**Authors:** Beshr Mosa Basha, Hazem Ahmad AlShikh, Nadia Abdullah AlAudah, Eman AlGhamdi, Fadhel Abbas Alherz, Khaled Subhi Essa Jebreel, Hazar Hadi Nahar AlFuraiji

**Affiliations:** Department of General Surgery, Dr. Sulaiman AlHabib Medical Group, King Salman Bin Abdulaziz Rd, Al Bandariyah, Al Khobar 34423, Eastern Province, Saudi Arabia; Department of General Surgery, Dr. Sulaiman AlHabib Medical Group, King Salman Bin Abdulaziz Rd, Al Bandariyah, Al Khobar 34423, Eastern Province, Saudi Arabia; Histopathology Department, Dammam Regional Laboratory, C3JM+73W, Ghirnatah, Dammam 32245, Eastern Province, Saudi Arabia; Pediatric Cardiac Intensive Care Unit, Saud Al Babtain Cardiac Center, Health Holding Company, 5443 King Khalid St, Ghirnatah, Dammam 32245-8443, Eastern Province, Saudi Arabia; Department of General Surgery, Dr. Sulaiman AlHabib Medical Group, King Salman Bin Abdulaziz Rd, Al Bandariyah, Al Khobar 34423, Eastern Province, Saudi Arabia; Department of General Surgery, Dr. Sulaiman AlHabib Medical Group, King Salman Bin Abdulaziz Rd, Al Bandariyah, Al Khobar 34423, Eastern Province, Saudi Arabia; Department of General Surgery, Dr. Sulaiman AlHabib Medical Group, King Salman Bin Abdulaziz Rd, Al Bandariyah, Al Khobar 34423, Eastern Province, Saudi Arabia

**Keywords:** gastrointestinal basidiobolomycosis, *Basidiobolus ranarum*, fungal infection, malignancy mimic, abdominal mass

## Abstract

Gastrointestinal basidiobolomycosis (GIB) is a rare fungal infection caused by *Basidiobolus ranarum* that predominantly affects immunocompetent individuals in tropical and subtropical regions and often mimics appendicitis, inflammatory bowel disease, or malignancy, resulting in delayed diagnosis and inappropriate management. A 38-year-old woman was previously treated for presumed Crohn’s disease or appendicular abscess without improvement and presented with persistent right-iliac-fossa pain, fever, palpable mass, and markedly elevated inflammatory markers. Computed tomography demonstrated a retrocecal inflamed appendix with peri-appendiceal collection, and clinical deterioration despite antibiotics and unsuccessful drainage necessitated laparoscopic appendectomy, followed by exploratory laparotomy and right hemicolectomy. Histopathology with special stains confirmed GIB, and itraconazole therapy resulted in progressive clinical recovery. GIB can closely mimic common inflammatory or malignant abdominal conditions, leading to diagnostic delay and repeated interventions; early clinical suspicion in endemic regions and prompt histopathological confirmation are essential to guide definitive surgical and antifungal management.

## Introduction

Gastrointestinal basidiobolomycosis (GIB) is a rare fungal infection caused by *Basidiobolus ranarum* that typically affects immunocompetent individuals and is frequently misdiagnosed [1, 2]. The organism, a filamentous fungus of the order Entomophthorales, resides in soil, decaying vegetation, and the gastrointestinal tracts of amphibians, reptiles, and insects, with infection presumed to occur mainly through ingestion of contaminated food or soil, although minor skin inoculation and insect bites have also been proposed [1, 3]. Since the first report in 1964, about 80 cases have been described worldwide by 2023, increasingly recognized over the past two decades and reported mainly in the Middle East, including Iran, Saudi Arabia, and Kuwait [1, 3]. Disease may remain localized but can progress to severe or life-threatening complications if diagnosis or therapy is delayed [4]. The colon and ileocecal region are most commonly involved, presenting with abdominal pain, fever, weight loss, or a palpable mass, often with eosinophilia [5]. Radiologic and endoscopic findings may mimic inflammatory or neoplastic disease [6]. Diagnosis relies on histopathology showing eosinophilic granulomatous inflammation with sparsely septate hyphae and the Splendore–Hoeppli phenomenon [7, 8], and management includes antifungal therapy with surgery for complications or diagnostic uncertainty [1].

## Case report

A 38-year-old Saudi female, presented with a 2-week history of progressive right-iliac-fossa pain associated with intermittent fever and a palpable mass. She had no chronic illnesses and a history of four cesarean sections. She had previously been hospitalized elsewhere for 2 weeks and treated with intravenous antibiotics for presumed appendicular inflammation or possible Crohn’s disease, without clinical improvement; details of that admission were limited, and no surgical intervention was performed.

On admission, Laboratory evaluation demonstrated leukocytosis (15.7 × 10^9^/L), neutrophilia, markedly elevated C-reactive protein (CRP) (91 mg/L), and prominent eosinophilia (2.48 × 10^9^/L; 16.8%). Contrast-enhanced computed tomography (CT) revealed a retrocecal fluid-filled appendix with a heterogeneous right pelvic collection inseparable from the cecum, associated with mural thickening and surrounding inflammatory changes (Fig. 1). Failed interventional radiology drainage prompted laparoscopic appendectomy. Gross examination of the appendix demonstrated ulceration and necrosis with hemorrhagic mucosa (Fig. 2). During the first postoperative week, she developed persistent fever, worsening inflammatory markers, and enlargement of the right-iliac-fossa mass despite meropenem therapy for extended-spectrum beta-lactamase (ESBL) *Escherichia coli* isolated from the appendiceal specimen. Repeat CT showed persistent cecal and ascending-colon inflammation without a drainable collection. Due to continued clinical deterioration, right hemicolectomy with ileocolic anastomosis was performed 10 days after appendectomy, revealing a large inflammatory cecal mass with pelvic collection and reactive lymphadenopathy (Fig. 3). Initial histopathology demonstrated a severe acute-on-chronic inflammatory process with microabscess formation, bacterial colonies, and a multinucleated giant-cell reaction. Grocott methenamine silver and periodic acid–Schiff fungal stains were negative, and there was no evidence of malignancy in 13 reactive lymph nodes. Despite negative fungal stains, persistent clinical suspicion of fungal infection prompted the initiation of empirical fluconazole therapy. However, the patient showed no clinical improvement and was readmitted 3 weeks later with recurrent right-iliac-fossa pain and a loculated intra-abdominal collection requiring CT-guided drainage (Fig. 4); tuberculosis polymerase chain reaction (PCR), mycobacterial cultures, and cytology were negative. Ongoing deterioration prompted expert re-evaluation of the hemicolectomy specimen using additional fungal stains, which revealed dense eosinophilic infiltration with thin-walled broad fungal hyphae and a Splendore–Hoeppli reaction, confirming GIB (Figs 5 and 6). Following confirmation of GIB, itraconazole therapy was initiated. At discharge, partial biochemical improvement was observed (white blood cell count 9 × 10^9^/L, CRP 140 mg/L, eosinophils 1.9 × 10^9^/L). After 1 month, inflammatory markers normalized with a significant reduction of eosinophilia to 0.8 × 10^9^/L, and the patient became asymptomatic with no evidence of recurrence on follow-up.

**Figure 1:**
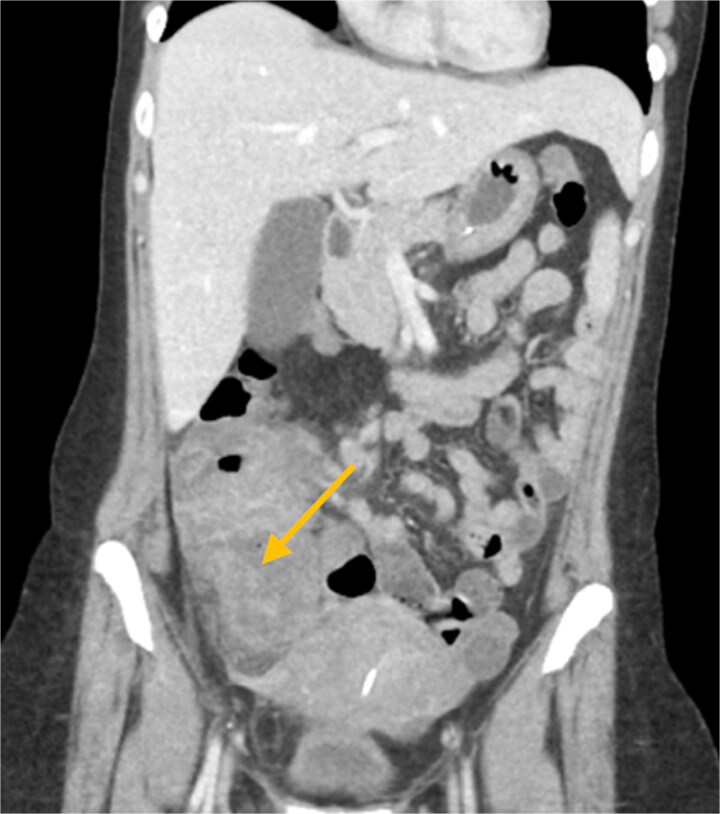
Contrast-enhanced CT scan of the abdomen and pelvis (coronal view); a right pelvic collection measuring 4.4 × 5.1 cm is seen inseparable from the cecum.

**Figure 2:**
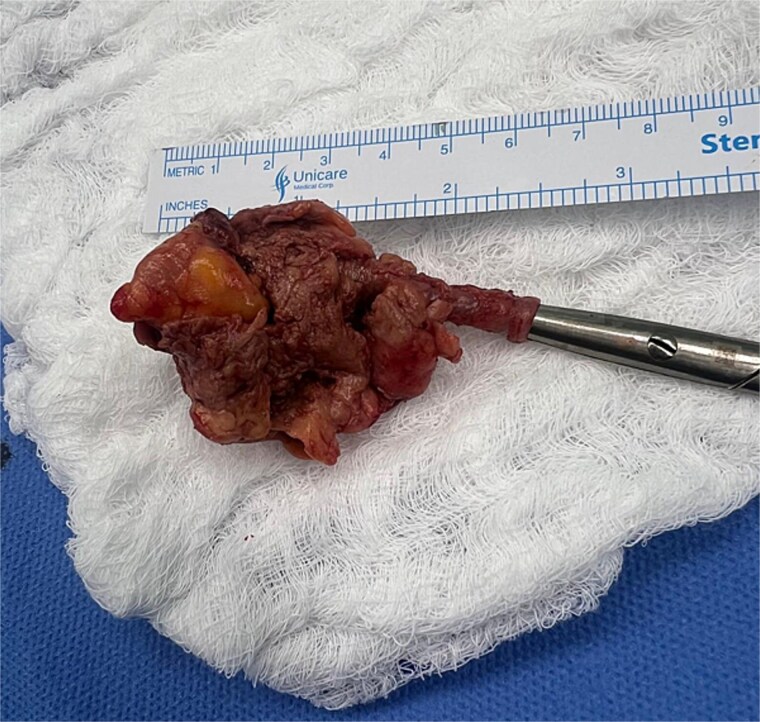
Appendix specimen; the specimen was received in two fragments measuring 6.2 × 3.5 × 2.4 cm in aggregate; the external surface appeared ulcerated and necrotic, while the cut surface revealed hemorrhagic mucosa.

**Figure 3:**
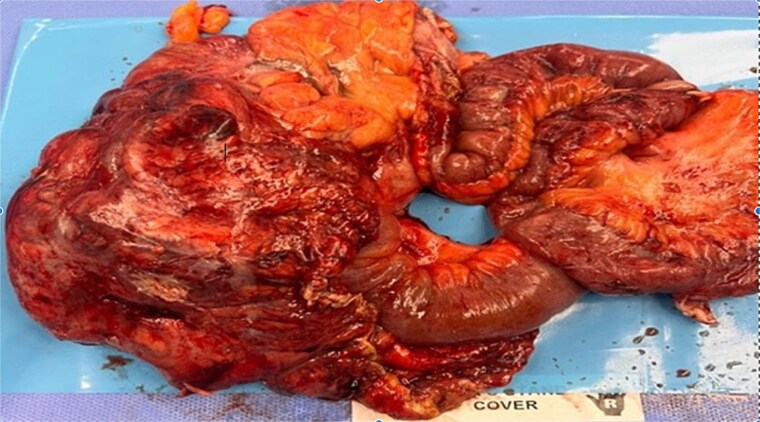
Gross appearance of the right hemicolectomy specimen; the specimen includes terminal ileum, cecum, and ascending colon with attached mesentery; a firm irregular inflammatory mass (phlegmon) is present in the retrocecal region, inseparable from the cecal wall, with indurated and edematous mesenteric fat.

**Figure 4:**
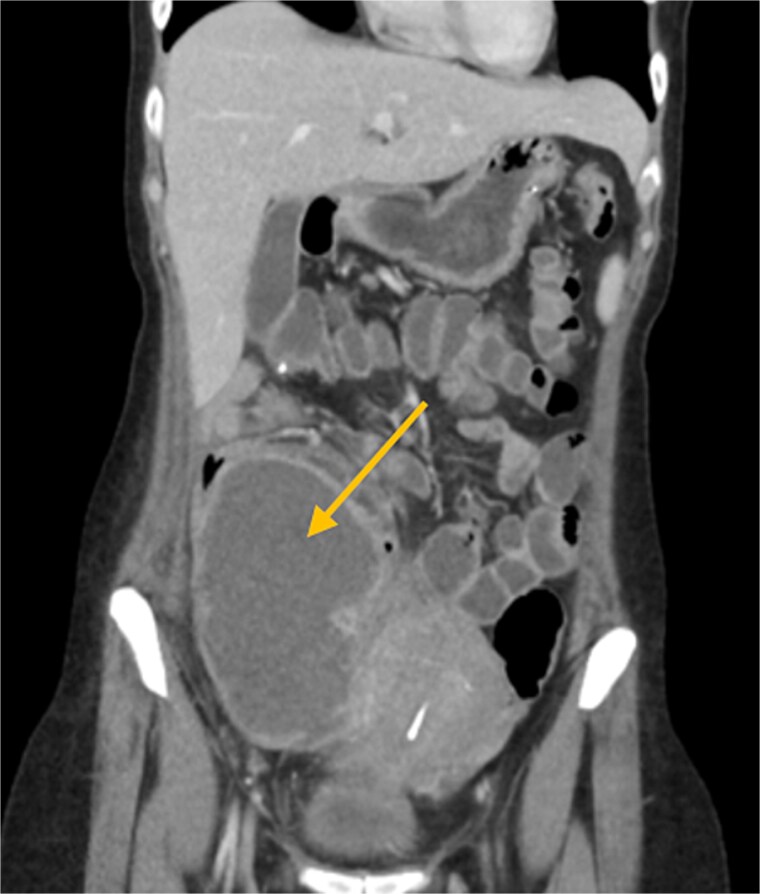
Contrast-enhanced CT scan of the abdomen and pelvis (coronal view); a large loculated intra-abdominal collection is demonstrated following right hemicolectomy.

**Figure 5:**
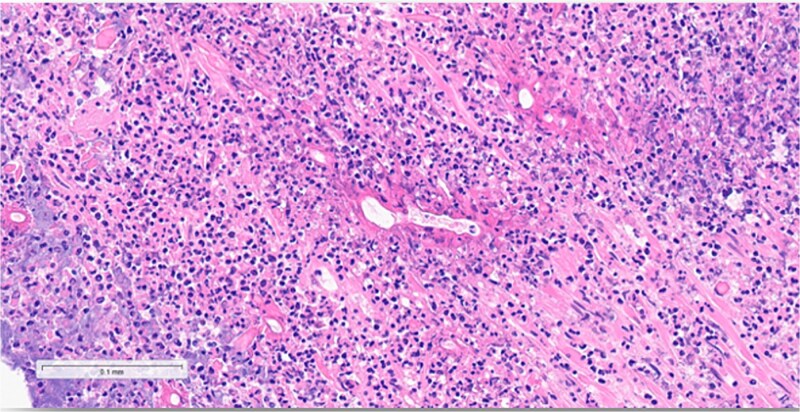
Histopathology of the resected colonic mass (hematoxylin and eosin stain); fungal hyphae are seen surrounded by eosinophilic cuffing consistent with the Splendore–Hoeppli phenomenon, with marked eosinophilic infiltration.

**Figure 6:**
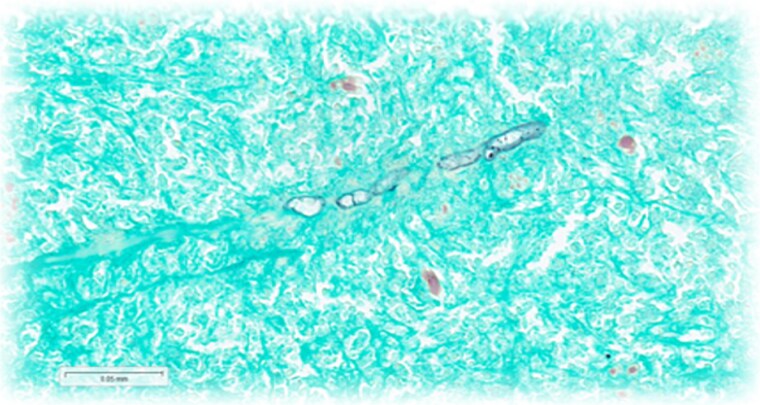
Histopathology of the resected colonic mass (Gomori methenamine silver stain); septate fungal hyphae are highlighted by the silver stain.

## Discussion

GIB is a rare but increasingly recognized fungal infection caused by *B. ranarum*, predominantly reported from tropical and subtropical regions, including Saudi Arabia [5, 6]. While classically associated with subcutaneous disease, gastrointestinal involvement represents a distinct entity that primarily affects immunocompetent individuals [2].

Clinical presentation commonly mimics appendicitis, inflammatory bowel disease, or colonic malignancy, creating significant diagnostic challenges [1]. The patient’s symptoms—right-iliac-fossa pain, fever, and a palpable mass—are consistent with previously reported cases initially misdiagnosed as appendicular abscess or Crohn’s disease [5]. CT demonstrating cecal wall thickening with surrounding inflammatory changes further contributed to uncertainty, reflecting the non-specific mass-forming inflammatory pattern frequently described in regional reports where patients often receive repeated antibiotics or empirical treatment for inflammatory bowel disease before GIB is considered [5, 6, 9, 10].

Peripheral eosinophilia, reported in most GIB cases, represented an important but initially unrecognized clue in this patient, persisting from admission despite broad-spectrum antibiotic therapy. In immunocompetent patients with mass-forming colonic lesions, eosinophilia should heighten clinical suspicion forz GIB [5].

Histopathologic evaluation remains the diagnostic cornerstone [7, 8], although early or superficial biopsies may fail to demonstrate fungal elements because hyphae are sparse or confined to deeper tissue planes [1]. This limitation was evident in our case, where the appendectomy specimen showed only acute suppurative inflammation with negative initial fungal stains, and definitive diagnosis required repeat staining and expert pathological review, as reported previously [1]. Re-evaluation demonstrated broad, thin-walled, sparsely septate hyphae surrounded by eosinophilic Splendore–Hoeppli material, characteristic of *B. ranarum* (Fig. 6) [8].

Empirical fluconazole was initiated when fungal infection was suspected but unconfirmed. Following histopathologic confirmation, therapy was transitioned to itraconazole, which shows the most consistent efficacy in GIB and produced rapid clinical and biochemical improvement similar to reported series [1, 6]. Combined surgical resection and prolonged antifungal therapy remain the cornerstone of successful management, particularly in patients with mass-forming disease, suspected malignancy, or failure of medical therapy alone [5, 6].

Overall, this case reflects the recognized clinical spectrum of GIB in immunocompetent patients and supports the effectiveness of combined surgical resection and itraconazole therapy described in regional and global literature [3, 5, 6]. In conclusion, GIB is a rare but increasingly recognized infection with nonspecific features that create significant diagnostic challenges and may mimic appendicitis, inflammatory bowel disease, or colonic malignancy in immunocompetent adults. Persistent eosinophilia, poor response to antibiotics, and mass-forming colonic inflammation should raise suspicion in endemic regions. Diagnosis depends on histopathology, sometimes requiring repeat or deeper sampling. Combined surgical resection and itraconazole therapy provide favorable outcomes, and increased clinician awareness is crucial for earlier recognition and improved prognosis.
